# Telecommunication Platforms for Transmitting Sensor Data over Communication Networks—State of the Art and Challenges

**DOI:** 10.3390/s16071113

**Published:** 2016-07-19

**Authors:** Kamil Staniec, Marcin Habrych

**Affiliations:** 1 Department of Telecommunications and Teleinformatics, Wroclaw University of Science and Technology, Wybrzeze Wyspianskiego 27, 50-370 Wroclaw, Poland; 2 Department of Electrical Power Engineering, Wroclaw University of Science and Technology, Wybrzeze Wyspianskiego 27, 50-370 Wroclaw, Poland; marcin.habrych@pwr.edu.pl

**Keywords:** wireless, cellular, ZigBee, electromagnetic compatibility (EMC), power supply

## Abstract

The importance of constructing wide-area sensor networks for holistic environmental state evaluation has been demonstrated. A general structure of such a network has been presented with distinction of three segments: local (based on ZigBee, Ethernet and ModBus techniques), core (base on cellular technologies) and the storage/application. The implementation of these techniques requires knowledge of their technical limitations and electromagnetic compatibility issues. The former refer to ZigBee performance degradation in multi-hop transmission, whereas the latter are associated with the common electromagnetic spectrum sharing with other existing technologies or with undesired radiated emissions generated by the radio modules of the sensor network. In many cases, it is also necessary to provide a measurement station with autonomous energy source, such as solar. As stems from measurements of the energetic efficiency of these sources, one should apply them with care and perform detailed power budget since their real performance may turn out to be far from expected. This, in turn, may negatively affect—in particular—the operation of chemical sensors implemented in the network as they often require additional heating.

## 1. Introduction

The intention behind this article is to provide readers with practical guide, derived from the authors’ own experience in this field, regarding some vital aspect on the sensor, telecom and IT parts, which—merged together—can make a successfully operating wide-area sensor network for environmental monitoring and modeling of threats.

In 1999, the Business Week [1] published 21 ideas that were believed to carry the greatest prospective potential in 21st century. These issues concerned all major aspects of our civilization, beginning with human and social-religious sciences through biology, Internet and energy sources. Among these ideas were cheap intelligent devices equipped with multiple sensors, scattered over a given area, thus creating a network of interconnected detectors allowing the monitoring of various parameters of the natural environment, technological processes (industrial automation, robotics, construction materials analysis, etc.), residential houses (temperature, humidity, carbon dioxide, etc.) or population clusters (e.g., the road traffic intensity measurement) [2,3,4,5,6]. While being intelligent and cheap (and therefore easily swappable), such networks can be installed in/on the ground, in the air, under water, inside/on the human body, and in vehicles and buildings. Beside the practical “peaceful” applications, the wireless sensor networks (WSN) also offer a wide range of services in military implementations, regarding reconnaissance, surveillance, tracking or guiding [7,8,9]. The latter application also entails aspects of the human life protection, e.g., in intelligent systems for explosive materials detection. Tests of such systems, such as Reverse Photo-Acoustic Spectrometer (REPAS), Ion Mobility Spectrometry (IMS) or Nuclear Quadrupole Resonance Spectroscopy (NQR) based on the ionic and acoustic spectrometry [10,11], were successfully carried out in laboratories, and they are now being widely exploited in war-afflicted regions.

There are multiple initiatives nowadays, providing proof of how significant it is to have a network of sensors or detectors rather than individual sensing devices. An example to mention is DARPA Sensor Information Technology (SensIT, Arlington, VA, USA), which is focused on the concepts of motion and direction tracking of vehicles and the monitoring of environmental conditions in preservation or natural parks. Some other large WSN projects include US NSF (National Science Foundation, Arlington, VA, USA), WINS or Smart-Dust [12]. The aim of the two latter projects is to integrate the functionalities of a sensor, a computational unit and a radio transceiver in a small and cheap module, easy to produce in considerable quantities. The Smart Dust project, in turn, has been developed on the basis of evolution in Micro ElectroMechanical Systems (MEMS) which allow constructing compact (with the volume on the order of 1 mm^3^), autonomous and mobile network nodes containing one or more sensors, beside computational and communication capabilities. On the communication side, these nodes are characterized by extremely low energy consumption (often supported by some built-in energy harvesting features), data transfer at the rate of a few kilobits per second and the ability to perform a self-organization process. The last feature means that in the initial stage nodes (in the Smart Dust project also called “motes”) are scattered randomly with no pre-defined topology or structure. With time they must be able to perform some simple routines to create a network with no external supervision, by finding the nearest neighbors and transferring the data via multi-hop transmissions (as will be discussed in greater detail in Section 3.3).

As a reader will notice, this article is not oriented towards future or cutting-edge technologies, but rather aims at providing an overview of existing communication technologies that lend themselves for use in transferring sensor data from where they originate to a desired location. An entire scope of the problem is covered: from hardware and software aspects through efficient methods of data storage as well as electromagnetic compatibility issues. All presented facts are a result of years of intensive research in this field and should be of interest to anyone concerned with efficiently transmitting sensor data over distances. Technical solutions described here have been successfully applied in a real working sensor network and presented in a manner clear enough to be easily replicated for the needs of any other sensor network, practically regardless of sensor types used there.

The author’s original contribution to the area includes:

- EMC (Electromagnetic Compatibility) investigations in Section 3.1 regarding the local segment.
- EMC investigations in Section 3.2 regarding the core network segment.

## 2. Wireless Sensor Networks (WSN)—Background and Concepts

Since the Internet has proven to be such an effective tool, it has been noticed that sensors, meters and detectors also make up a network “society” and participate in the network traffic on equal rights as their human peers. The data they generate are also often of no less importance than of those generated by man; they are often core data for maintaining life quality, control over industrial processes or environmental monitoring. Those devices can communicate either with a human (operator) or directly with other devices. The latter method is known under the abbreviation M2M (Machine-to-Machine), constituting one of the paradigms of the Internet of Things (IoT), i.e., such a form of our worldwide web where machines are partners of humans. As an aside, it is worth noting that many scientific institutions that are dealing with this topic openly admit that they have started to consider this problem having read a 1982 “Observation on the Spot” (Polish “Wizja lokalna”) novel by Stanisław Lem, in which the author showed a world where basic life necessities (food supply, security, cleaning, environmental monitoring, etc.) were performed by an intelligent network of integrated sensors, which—relieving humanity of such time-consuming chores—enabled humans to stop thinking about down-to-earth matters and start conquering outer space. Thirty years after the publication of the book, the technological development seems to correspond to the vision presented in the novel. The authors believe that future belongs to “systems”, understood as sets of devices that communicate with each other, thus creating a kind of diffused intelligence. Only then can the sensor information—especially that related to environmental monitoring—release its full potential in terms of providing a representative image on the quality of air, soil or water to render a holistic, instead of discrete point, image of situation.

**Figure 1:**
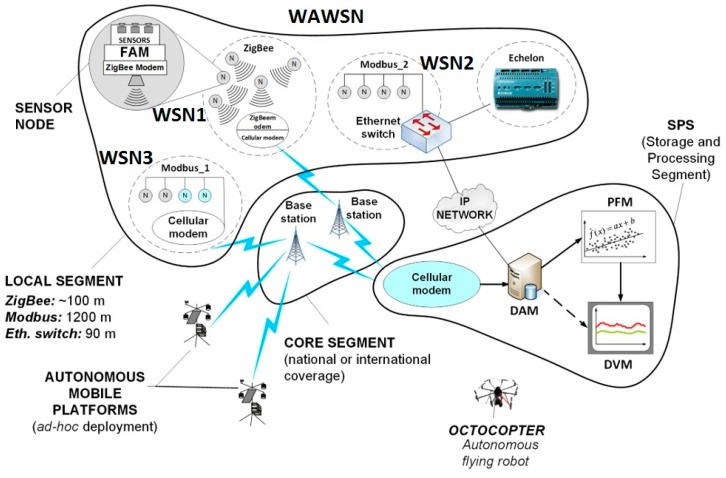
A block architecture of a wide-area sensor communication system.

There is no single “good-for-all” communication technology available that could be advised to use as a telecom platform for a sensor network. However, the solutions that exist are already mature and fitted for different needs [13]. Therefore, the article will show how a combination of various technologies can be utilized to create a flexible architecture of a robust and scalable network for transmitting data from arbitrary sensor types to a desired location. Regardless of specificity of particular applications, in the most general form the purpose of a sensor network is to assure a bi-directional data transfer between the management center and multiple sensors scattered in various locations depending on a type of the monitored environmental phenomenon. The final structure of the network, viewed not only as a transmission network but also as a whole system for the environmental monitoring, is shown in Figure 1. As can be seen, the network consists of three basic components: the Local Segment, the Core Segment—both related to the communication technologies—as well as the Storage and Application Segment (SAS)—related to methods of storing the measured data, their visualization and processing. The local segment solutions refer to the notion of Wireless Sensor Networks (WSN) and are usually constrained to limited geographical areas. The entire structure comprising multiple WSNs (three in Figure 1) is therefore referred to as a Wide-Area WSN (WAWSN).

In the local segment, particular sensors are viewed to be the outermost elements of the system as they directly interface with a given sensed phenomenon (which includes electromagnetic field/smog, noise/acoustic smog, organic and inorganic gases, industrial and biological waste e.g., microbiological water control, gram-negative bacteria, etc.). It should be noticed that sensors themselves are sometimes an issue, especially regarding their powering (see Section 4 for more details), although now the attention will be paid to the data transmission aspects only. After measurement and initial pre-processing, the analog output signals (i.e., the output current, voltage or resistance) need firstly to be adapted in the Format Adaptation Module (FAM) (see Section 2.1.2 for details) to the form acceptable by a communication system, which will further on be referred to as “local”. A recommendable candidate technology for this segment is the ZigBee system basing on the IEEE 802.15.4 specification [14], described in more in-depth in Section 2.1. It should be noted, however, that there exists a plethora of other solutions available to create WSNs in the local segment, depending on the radio range, throughput, bandwidth, price, mobility, etc. These include systems such as: Wireless Hart, 6LoWPAN, RFID (Radio Frequency Identification), RF4CE, SERCOS, BLE (Bluetooth Low Energy), NRF24L01, NFC (Near Field Communication) and many others. ZigBee, however, appears to be the most mature of all others in terms of suitability for telemetric applications, due to its: energy savings operation modes, relatively low throughput, high robustness to interference/multipath ([15,16]) and effective built-in routing and multiple access mechanisms. 

One of the ZigBee devices will have to be a dual-technology device, collecting data from multiple other ZigBee modules and passing them on to the core segment via a GPRS modem (see Modbus_1 module in Figure 1). Alternatively, sensor boards can be connected directly—through FAM—to the concentrator. However, one needs to remember that radio solutions are vulnerable to various sorts of interference (more on this in Section 3.1 and Section 3.2). Hence, if a wired connectivity is available it is advisable to take advantage of it and connect a sensor directly to an Ethernet switch (see Modbus_2 module in Figure 1) and from there to the Data Acquisition Module (DAM). As one can also notice, it is relatively easy to connect several sensors in series by means of the ModBus technology with a simple Ethernet cable. This issue as well as the other techniques mentioned in this section will be described in greater detail in later chapters.

**Figure 2:**
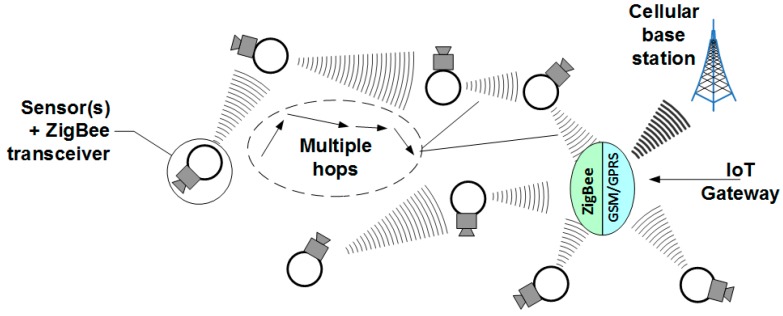
The local segment with multi-hop transmissions between sensor modules prior to reaching the IoT Gateway.

Naturally, these scenarios are not universal solutions but they have been verified to work well and provide an almost unlimited extent of the sensing area due to the widespread coverage of current cellular networks and/or IP network accessibility. The ease of deployment must also be appreciated since the sensor network operator is alleviated from the transport segment, which is totally the issue of a cellular (or an IP services) provider. In the IoT nomenclature, the cellular modem located on the interface between the sensor network part and the cellular (or IP cloud) part, can be considered as an IoT gateway device. Gateways, in general terms, may be used of two purposes: either for simple translation between WSN protocols (here: ZigBee, see also in Figure 2) and the cellular/IP protocol or as devices that—beside protocols translation—also provide with and “Edge Intelligence” (EI) to it. The EI term means that prior to passing messages that arrive from the WSN side towards the IP or cellular cloud, the gateway device will perform some computations (that require ability to interpret the WSN information) concluded by decisions whether or not the information should be carried forward, discarded or retained. The latter means that the gateway may decide to wait for more readings to flow in from other WSN sensors in order to either confirm the already received message or to perform their aggregation and send to the IP/cellular cloud only a summary report instead of conveying the entire traffic generated on the WSN side. Such a behavior is strongly recommended for the sake of saving the IP or cellular networks capacity from excess transmission of redundant sensor data (see also [17]). As is often the case, however, the price to be paid for enhanced logic is that of increased energy consumption and greater financial costs per gateway device. Fortunately, the IoT market has spawned numerous gateway solutions that come from multiple different vendors, not necessarily major ICT players, that perform these EI functions natively. If only protocols translation is needed, however, WNS/cellular modems should suffice.

On the other end of the network, the data collected from sensors with different frequency and volume depending on a sensor type, is received by the GSM/GPRS (Global System for Mobile Communications/General Packet Radio Service) modem—the first element in SAS—and stored here in fast, redundant and safe databases (DAM). They can be further passed on to the Processing and Forecasting Module (PFM) for trends extraction, anomalies detection and forecasting the sensed phenomena behavior based on their measured history. Eventually, data can be either accessed directly (in their raw format) or through a Data Visualization Module (DVM) for the presentation of only some desired aspects according to the end-user’s discretion.

As was already mentioned in Section 1, the article presents and discusses technologies (both wired and wireless) advised for use in each communication segment, by briefly introducing their specifications, proposals of practical implementation, technical limitations and electromagnetic compatibility (EMC) issues that need to be reckoned with in the design stage in order to ensure operational robustness and reliability. All information provided in the paper is a result of experience obtained by the authors during their work on a national-wide communication network for monitoring the environmental.

In later sections, the term “concentrator” will often be used to refer to the whole module consisting of the following parts: the physical and chemical sensors connected in series, the FAM and the GPRS modem. Throughout the article the term GPRS (General Packet Radio Services) will be used generally in reference to any technique for packet transmission over cellular networks, including EDGE, UMTS, HSPA(+DC) or LTE. The reason why telemetric networks can be formed with the use of seemingly old-fashioned technologies in the backhaul (such as GPRS) is due to the usually small volumes of data generated by sensors (tens of kilobits per second) as compared to the throughput capabilities offered by modern cellular technologies (e.g., LTE) that allow hundreds of megabits per second of data rate. This means that the choice of communication technique in the core segment should be fitted to real required throughput and reliability (coverage) rather than the novelty of solution. Doing otherwise may lead to a gross underuse of available transmission bandwidth. Typically, older (slower) systems also feature a much more widespread service availability (i.e., radio range) than the newest ones since the latter are often deployed in highly populated areas for fast investment return and high revenue, leaving “emptier” spaces uncovered. This may be of some problem if one accepts the sensor network operational range as a crucial planning criterion.

### 2.1. The Local Segment

#### 2.1.1. ZigBee (IEEE 802.15.4 Specification)

Out of the multiplicity of candidate solutions for effective creation of sensor networks is the ZigBee standard based on IEEE 802.15.4 specification [14]. It defines a low-cost, low-range, power-saving system allowing data rates—depending on the frequency band, of 20, 40 and up to 250 kb/s (theoretically, although less so in practice, as discussed in Section 3.3) and almost inexhaustible number of network nodes (typically 16 bits allocated for addressing or 64 bits using extended addresses). ZigBee has been defined in three separate bands with 27 distinct channels defined therein, as given in Table 1. Depending on the application requirements, ZigBee devices may operate in either of two topologies: the star topology or the peer-to-peer topology. In the star topology, communication is established between devices and a single central controller, called the Personal Area Network coordinator. The peer-to-peer topology also has a PAN coordinator; however, it differs from the star topology in that any device may communicate with any other device as long as they are in range of one another. Peer-to-peer topology allows more complex network formations to be implemented, such as mesh networking topology. In practical situations, of course, one will deal with a combination of these two, as shown in Figure 2 (the figure, in fact, shows ZigBee part of the Local Segment in Figure 1).

The ZigBee technology is suited for ad hoc, self-organizing, and self-healing networks, which means that one’s individual devices are turned on, they will automatically organize themselves in a network structure by exchanging beacons and replies. It is a very attractive feature of this technology since it relieves the persons involved in measurements of the burden associated with the network planning—the measured data will be transferred via multiple “hops” from a source node (i.e., a sensor + ZigBee radio transceiver) to the concentrator (i.e., the GSM/GPRS modem). Interested readers are advised to refer to [18,19,20,21,22] where limitations of these mechanisms are discussed and improvements are suggested.

Applications such as industrial control and monitoring, wireless sensor networks, asset and inventory tracking, intelligent agriculture, and security would benefit from such a network topology. A peer-to-peer network can be used but it may also allow multiple hops to route messages from any device to any other device on the network. Such functions can be added at the higher layer, although they are not part of the 802.15.4 standard.

Two different device types can participate in an IEEE 802.15.4 network: a full-function device (FFD) and a reduced-function device (RFD). The FFD can operate in three modes serving as a personal area network (PAN) coordinator, a coordinator, or a simple device. An FFD can talk to RFDs or other FFDs, while an RFD can talk only to an FFD. The RFD is intended for applications that are extremely simple, such as a light switch or a passive infrared sensor; they do not need to send large amounts of data and may only associate with a single FFD at a time. Consequently, the RFD can be implemented using minimal resources and memory capacity. Real devices standing behind these abbreviations will also differ in price, with RFDs being cheaper and FFDs being more costly.

#### 2.1.2. Modbus—GSM/GPRS Modem

**Figure 3:**
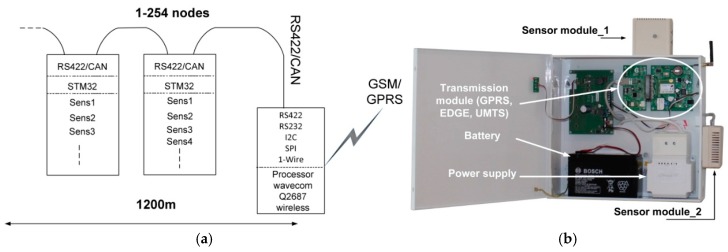
A ModBus network concentrator module: (**a**) a schematic view of transmission chain; and (**b**) a photo of a real concentrator including power supply.

The application of the ZigBee technology in combination with the battery power supply (most often the case) means a short life time of the sensor module. Hence, another variation of the concentrator has been designed (M2G (Modbus2GSM)), to which sensors are connected by RS422 or CAN network, using ModBus RTU as a transmission protocol. In this way, scalability in the local segment has been achieved allowing up to 254 sensor modules connected to a single concentrator. Additionally, the same cable is shared for both energy supply and data transmission. The sensor module is equipped with its own ARM Cortex-M3 microprocessor, two interfaces (in compliance with the ModBus standard) and several gas sensors (depending on needs). A block diagram of such a concentrator is presented in Figure 3a. The real concentrator should obviously also include a power supply and a casing isolating it from adverse external factors (such as adverse EM radiation, inadvertent violation, etc.), such as shown in Figure 3b.

**Figure 4:**
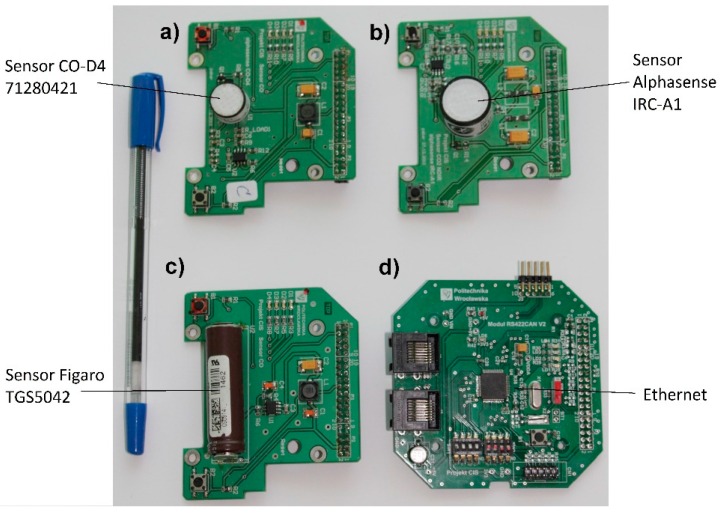
A top view of: (**a**–**c**) examples of FAM module with sensors; and (**d**) a microprocessor module.

The sensor module construction comprises two circuits (Printed Circuit Boards):

- a Format Adaptation Module FAM (Figure 4a–c); and
- a microprocessor module (Figure 4d).

The microprocessor module, universal for all sensor nodes, is responsible for the ModBus operation. It performs basic mathematical calculations and, by its Analog-to-Digital and Digital-to-Analog converters (ADC and DAC, respectively), samples the signal processed by the FAM module. The FAM module (consisting of amplifiers, filters, reference voltage and/or current sources, etc.) in turn, is responsible for the initial analog processing of the sensor signal in accordance with its application note. Examples of FAMs shown in Figure 4a–c demonstrate three types of sensors different by the signal returned type (voltage, current, conductivity, etc.). Moreover, some sensors may require some additional processing for calibration purposes. FAM modules are therefore indispensable to process sensor readings (whenever required) and/or convert their outputs to a unified data format used in ModBus.

Such a construction allows having only one microprocessor module and as many FAM modules as there are different sensor types used. The modular architecture allows one to scale the platform practically infinitely by adding new sensors, which—after their installation on an appropriate FAM module—can then be easily integrated with the microprocessor module.

The concentrator module may also be equipped with an additional Ethernet port. Such an implementation offers a redundant data transmission technology on the access segment level. The concentrator may operate both in the Ethernet and GSM networks simultaneously or separately. The Ethernet interface is integrated into the Q268x modem firmware as another medium to be considered in the sensor network. Other concentrator features on the higher TCP/IP layers (from the link layer upwards), including the software and the protocol, have been preserved.

### 2.2. The Core Segment

It can be useful to divide the core segment into two distinct functional components:

- server infrastructure; and
- transport network.

**Server infrastructure**. The DAM module is one of the key components of the sensor system. It is responsible for receiving data gathered by the concentrators and their correct insertion into databases. Depending on the application, DAM has to meet the following requirements:

- immunity to the database inaccessibility (local database buffering);
- appropriate scalability; and
- a high single transaction handling efficiency (a transaction is understood as operation of feeding data into a database).

The http is proposed as the application protocol for the following purposes: system unification purposes (Internet Protocol (IP) packet transmission through the Internet), the use of well-known technical and infrastructure solution that have proved to work well with major Internet portals such as Facebook or Twitter. Two aspects require investigation:

- minimization of the server load through the web server; and
- scalability by building a farm of web servers one of which functions as a load-balancer.

**Figure 5:**
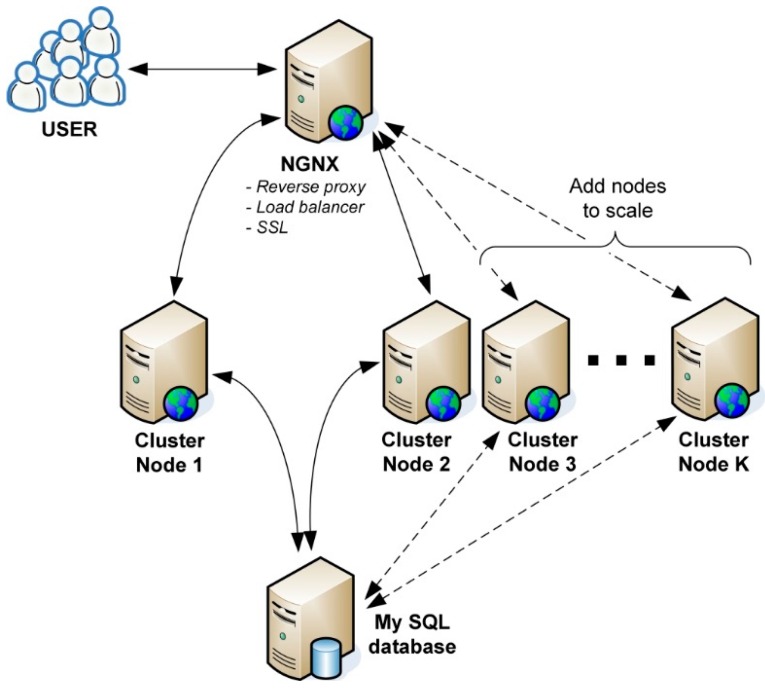
An example solution for the Web cluster.

The use of NGINX (a proper name pronounced as “engine X”) in the web cluster, as shown in Figure 5, instead of the commonly used Apache, allows achieving both these goals. NGINX is a powerful high-performance web server that provides a complete http/https server in addition to a reverse proxy, content cache and load balancer.

The “user” symbol in Figure 5 denotes a client (which can be the web browser of an end user watching a web-site with diagrams) and/or a concentrator. New connections arrive at a so-called load-balancer, which is, in fact, a web server redirecting this connections to one of the K available working Web servers (or “nodes” in the figure). This enables balancing the load and increases the service availability. If, in turn, the system requires increased efficiency, it suffices to add new web servers do the load-balancer configuration. Each of the working servers processes the task by reading or writing appropriate data in the database. The connection between the client/concentrator and the load-balancer is encrypted with TLS/SSL protocol, whereas the redirected traffic in unencrypted. In this way, the security is not lessened (provided that the servers are physically secured) while the overall efficiency is remarkably improved. The failure of one of the working Web servers does not affect the entire cluster operation, which is a noteworthy value of the proposed solution.

**Transport network**. The proposed construction of the sensor modules and concentrators has been introduced in Section 2.1.2. Depending on the preferred data transmission method (in terms of either by accessibility or applicability), there are two options to choose between:

- wired connection in the Ethernet technology; and
- wireless connection using the GPRS technology (see extended definition of this term in Section 2).

In general, the cellular technology allows for packet transmission utilizing Internet Protocol (IP). Both proposed concentrator implementations use the IP protocol in the network layer. As an application layer protocol, http and its encrypted (with TLS/SSL) version https are recommendable.

The Ethernet connection has one significant virtue—if available, the transmission is cost-free because the permanent network access is usually fixed and constant. In cellular networks, on the contrary, transmission is billed based on the volume of transmitted data (in each direction separately) in operator-dependent units of, e.g., 10 kb. In other words, the more data sent over cellular, the more costs generated. In order to assure improved security level (beside TSL/SSL encryption), cellular network providers offer a so-called dedicated private APN (Access Point Name) network. With this solution, the SIM cards will work within a separate (virtual) network, accessible only to selected clients. No other SIM card will have access to it unless specifically added to the list, which creates an extra “physical” connection security, analogous to the VLAN Ethernet technology.

Regarding the financial side of investment in the technologies described above, prices of hardware and software components will obviously differ by country. However, some general estimations can be made based on a survey of vendor offering their products worldwide and presented briefly in Table 2.

### 2.3. The Storage and Application Segment

The last component is the database in which all measurement data are stored (for visualization, analysis and prediction of state or threats). It is therefore significant to make it coherent, efficient (in terms of capacity or data access time) and always accessible.

**Figure 6:**
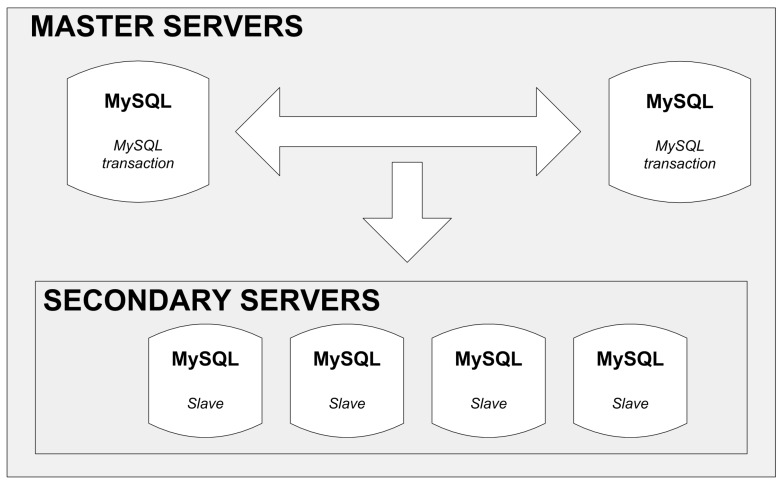
An example solution for the Web cluster.

A recommendable open-source product to consider is MySQL (Structured Query Language) database. It is a classic relational base supporting the SQL language and transactions in compliance with ACID (Atomicity, Consistency, Isolation and Durability) paradigm [19], and has been well-tested in multiple ICT (Information and Computer Technology) projects as well as Internet portals (e.g., Facebook). In the simplest installation version, the database consists of a single server. However, the basic limitation of his configuration is a complete lack of redundancy: the failure of a disc or a system breakdown renders the whole database inaccessible. The data replication by means of installing a back-up server can be a recommendable solution to this problem, as shown in Figure 6.

In this solution, there are two main database servers (Master), only one of which is active at a given moment. The other one serves as a backup. The data are also replicated in secondary servers from which they can only be read. The replication process is as follows.

- New data (added to the database) or data modified in the base are processed in only one master server.
- Every specified time quantum, data are replicated to the backup server and a group of secondary servers (the time between successive replications is on the order of a few seconds).
- Data can only be read (e.g., for visualization) from one of the available secondary servers.

A basic weakness of this solution is the lack of automatic switching from the main to the backup master server in case the former fails. Moreover, there is no certainty that the data are coherent. Thus, it is possible that the new data written in the main master server (before its breakdown) have not been replicated. This may happen because the replication is performed not on the rows level (record addition or its modification) but on the table level every time quantum. It is a major drawback making this server configuration inadequate to run online services with SLA (Service Layer Agreement) above 99.9%.

**Figure 7:**
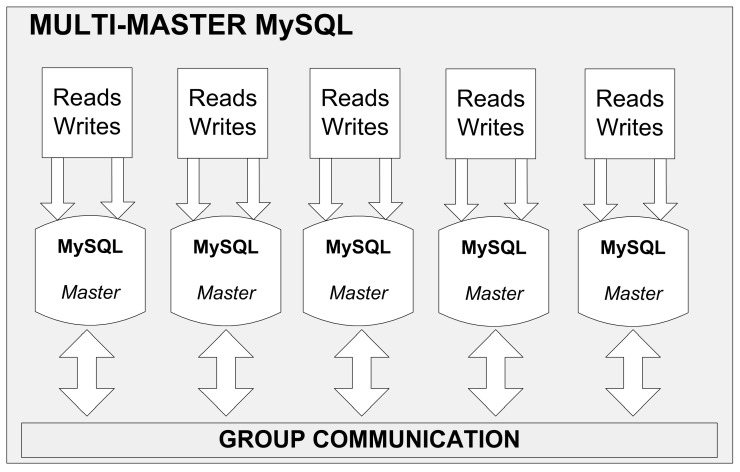
The operation of Galera replication.

By far the best and commendable solution is to configure replication on a single database row basis. Such a replication is offered by the most up-to-date MySQL database server installations like NDB or Galera. The former requires large RAM memory, which is where tables are stored (not on discs), and is dedicated to telecom providers. Galera replication is cheaper but is still equally. The database cluster (see Figure 7) consists of N master servers working concurrently. They are all on the same level of hierarchy and perform identical operations on the database. Therefore, whenever an *i*-th server writes data (after transaction completion), it is immediately available on an any other server, which prevents data incoherence. The scalability consists in adding new servers to the pool. The servers can be run on virtual and/or physical machines, located in situ or in a data center since replication in the Galera allows building an effective database cluster (viewed as a single database) scattered between different physical locations (i.e., server rooms). The failure occurred in one center does not impair the entire data base system in any way but its correct operation requires availability of at least two master servers.

## 3. Issues and Challenges in WSN

Keeping in mind all advantages offered by radio technologies for sending sensor data over distances, one should also be aware of some electromagnetic compatibility (EMC) issues that may arise during wireless network exploitation. In some cases, interference is originated since multiple systems operate in the same unlicensed frequency band (see Section 3.1). In other cases (see Section 3.2), imperfections in the construction of radio modules operating in licensed bands may inject undesirable disturbances into different bands than those used for the desired transmission. These impairments in cellular modems, although easy to remove by means of known EMC techniques, may often be disregarded when assembling a customized device such as the one presented in Figure 3b. For this reason, it is highly recommendable to perform EMC tests for emissions and immunity in an accredited laboratory. Problems with unintended out-of-band emissions may appear from the following two major sources.

- Application of wireless modules (either in the local segment, e.g., ZigBee or Bluetooth, or in the core segment, e.g., cellular modems) from questionable or unknown sources enticed by their low prices.
- Undesirable radiation originated not necessarily from the radio modules themselves as rather from the whole sensor units (here referred to as “concentrators”), including sensors, FAMs, batteries and radio module(s), such as one in Figure 3b. The rationale behind this approach is based on the observation that unpredictable and unwanted emissions may also arise due to current flow in wired interconnections between these components and be effectively radiated from apertures in the casing.

Regardless of the EMC problem source, it is a recommendable practice that whole sensor devices—not just separate components—be tested for EMC compliance.

### 3.1. EMC in the Local Segment

**Figure 8:**
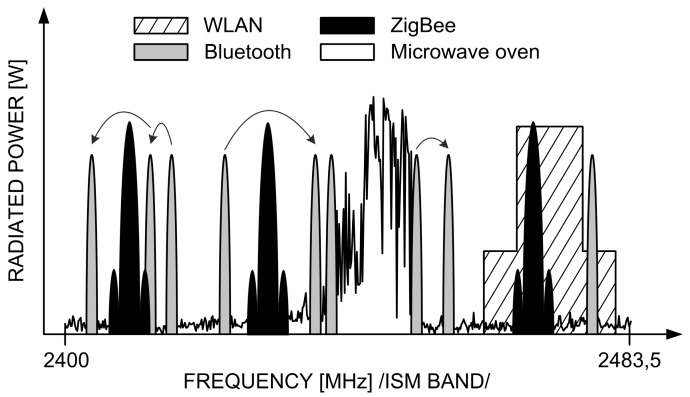
Technological congestion in the ISM band [16].

ZigBee technology is designed to operate in a highly congested electromagnetic environment. There are multiple other transmission techniques and systems that share the common Industrial, Scientific and Medical (ISM) band spans between 2400 and 2483.5 MHz. These systems include (but are not limited to) the Wireless Local Area Networks (WLAN) and Bluetooth, as major users. To complement the interference situation, one should also remember that microwave ovens are powerful sources of EM radiation in the ISM band, too. A typical scenario in the frequency domain is shown schematically in Figure 8.

In all EMC experiments presented herein, ZigBee devices were set to the maximum Equivalent Isotropic Radiated Power (EIRP = 20 dBm) in order to make sure that there is no more room for maximizing the ZigBee signal and the only remedy against interference is one stemming from the repetitions mechanism natively employed in the IEEE 802.15.4 specification.

**Figure 9:**
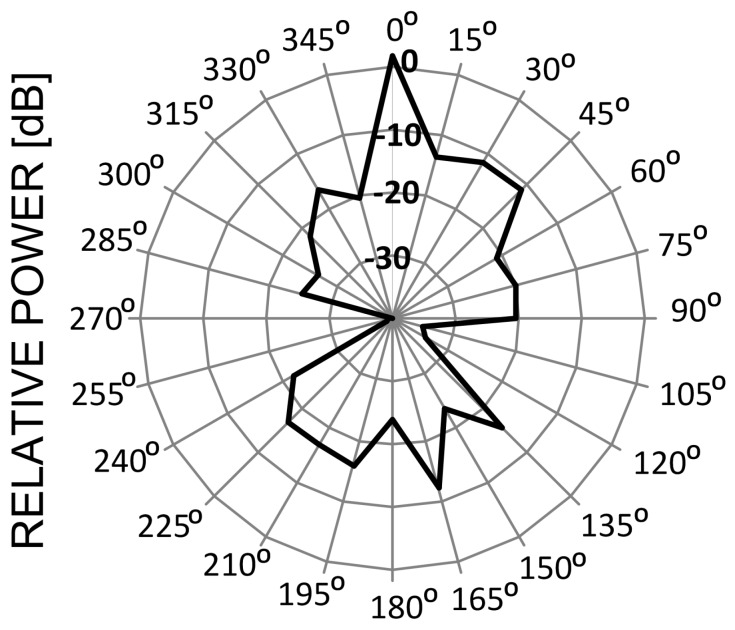
Radiation pattern of the microwave oven [16].

All these systems depicted in Figure 8 differ in the way they transmit signals in frequency and time. For instance, in Bluetooth 1 MHz-wide channels hop 1600 times a second in a pseudo-random fashion across the entire ISM band. Frequency channels in WLAN, ZigBee and microwave oven—once defined—preserve constant positions on the frequency axis and are different by shape and width: 20 (22) MHz, 2 MHz and ca. 30–40 MHz, respectively. As for the antenna radiation pattern which tells how a given device radiates in different directions, communication systems (ZigBee, WLAN, and Bluetooth) are usually omni-directional, which means that they radiate equally in all directions. As for the microwave oven, in turn, the strongest radiation is generated from the oven front and its back, whereas is attenuated on its sides (blocked by the solid metal enclosure), as shown in Figure 9 (0° direction represents the oven front).

Now, a few diagrams will be presented [20,21] that show a distance dependence between ZigBee devices and WLAN device as well as the microwave oven. As a metric of interference, the authors assumed the Packet Error Rate (PER) that is a percentage indicator of the number of lost packets (or received with errors) due to EMC issues.

**Figure 10:**
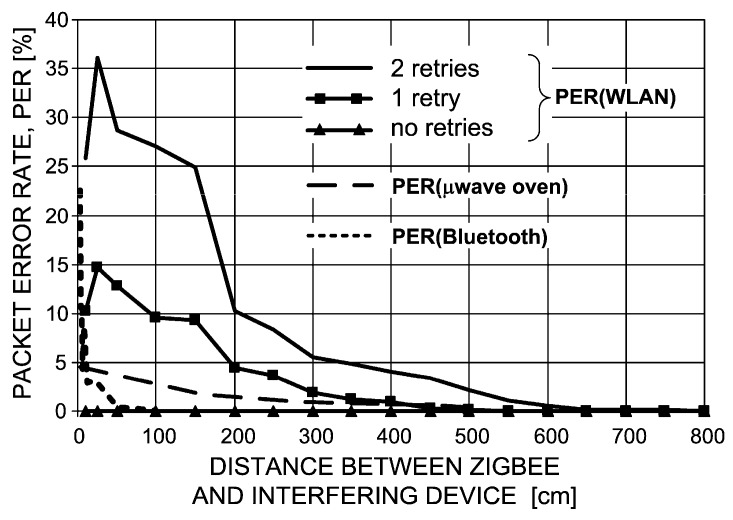
ZigBee Packet Error Rate vs. distance from WLAN, a microwave oven and Bluetooth [16].

As can be seen in Figure 10, PER in a ZigBee device diminishes to almost zero when separated from a WLAN device by a distance of six meters, with the WLAN card set to radiate at its maximum EIRP, i.e., 100 mW. However, even if this condition cannot be met for some reasons, there is one intrinsic feature incorporated into most ZigBee implementations that helps alleviate the interference problem, namely retransmissions (or retries).

With this mechanism turned on, the ZigBee transmitter will retry, for a user-defined number of times, sending a given packet until an acknowledgement with positive reception is received. As can be seen, PER reacts very dynamically to this parameter: with as few as two retries, ZigBee transmission is practically insensitive to interference from WLAN systems (PER ≈ 0%). 

As regards interference from a microwave oven, the dashed line in Figure 10 shows that a 5% packet loss can be expected when ZigBee is located in a direct vicinity of the running microwave oven door. As the distance increases, PER exponentially fades away to negligible values at ca. 5 m.

Finally, Bluetooth appears to have the most benign effect on ZigBee operation. Its influence is significant only at immediate proximity on the order of 0–50 cm. Initially, PER peaks at 23% and swoops abruptly tens of centimeters further away, down to 2%–3%. At one meter of separation between both systems, interference from Bluetooth can be considered as non-existent. Such an effect should be attributed to the specificity of Bluetooth transmission where each 1 MHz-wide channel dwells on a given frequency for only *t_dwell_* = 625 μs and then hops to another pseudo-randomly chosen frequency. With the total available 79 such channels in the ISM band (4.5 MHz is subtracted from the net 83.5 MHz spectrum defined in Figure 9, as guard bands) and the ZigBee channel width of 2 MHz, one can easily calculate the probability that over a given period *t_dwell_* ZigBee and Bluetooth channels will overlap to be equal 2.53% (i.e., 2/79).

For the sake of comparison, with the other analyzed systems (WLAN, microwave oven) this probability is 100% since once overlapped, channels remain fixed in the frequency domain for the entire transmission period.

### 3.2. EMC in the Core Segment

**Figure 11:**
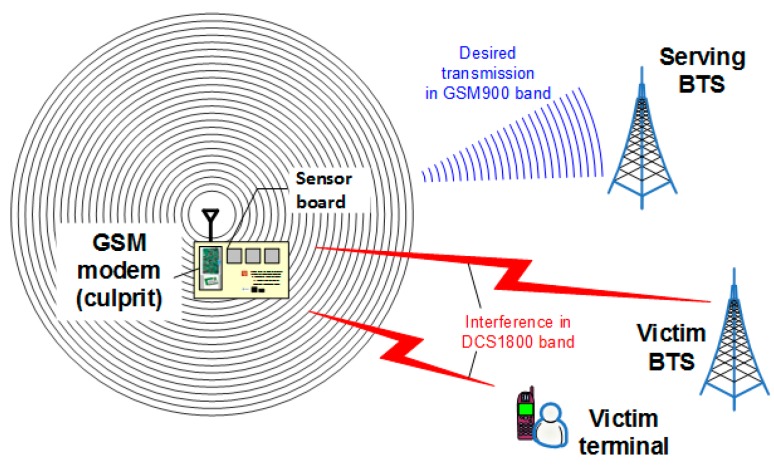
Interference caused by the GSM modem (concentrator) in 900 MHz band to a terminal and a BTS in 1800 MHz band.

As was already mentioned, in some cases, interference is caused by imperfectly constructed physical devices that produce radiation in unwanted frequency bands, thus interfering with other radio systems residing there. Since the core segment consists of equipment operating in licensed cellular bands (refer to Figure 1), it is treated as a regular cell phone and is subjected to the same co-channel interference mechanisms. Interference to other in-band GSM terminals and base stations (BTS) is not an issue since the core system by itself prevent transmission on the same frequencies and time slots within a given cell. Therefore, the authors will rather focus on interference caused by the concentrator to other cellular system users and BTSs that operate in a different frequency band, as in Figure 11.

**Figure 12:**
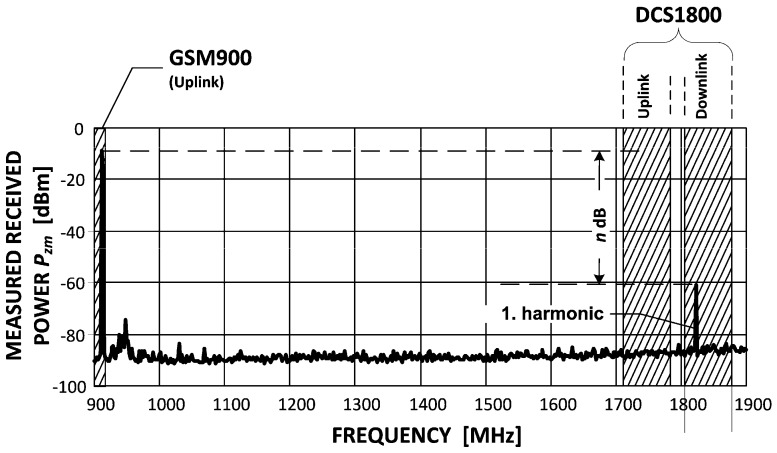
Radiated emission from the core GSM modem (core segment): the wanted signal and the undesirable 1st harmonic.

One will begin with showing the radiated power vs. frequency from the GSM modem. For high output power settings, in many commercially available modems, one may expect that due to the amplifier non-linear characteristic a relatively strong 1st-harmonic signal (n decibels weaker than the desired signal) will be generated as in Figure 12.

Unfortunately, this unwanted radiation—though derived from a 900 MHz band operation (i.e., GSM900)—falls into another GSM band. Uplink (UL) and downlink (DL) terms mean transmission from the terminal to BTS and vice versa, respectively. In other words, both—terminals and BTSs—may be victims to interference from a GSM modem (the concentrator) transmitting sensor data. 

The strength of this disturbance is naturally mitigated by attenuating factors such as vegetation or buildings; however, for the purpose of EMC investigations, the worst-case scenario should be considered in which free-space (i.e., an unobstructed line of sight, LOS) propagation conditions are present. In LOS, the radio signal pathloss *L_FS_* is given by:

(1) $$L_{FS}=32.45+20\log(f_{MHz})+20\log(d_{km})$$

It is now left to calculate the safe distance *d_safe_* from both GSM terminals and BTSs. Investigations, however, need to be performed separately since different parameters apply in each case. Basic factors that need to be considered are: the sensitivity power *P_sens_*, the correction COR (20 dB for terminals and variable for BTS) and the Signal-To-Interference Ratio (SIR); all specified in a respective norm [22] which addresses co-channel interference aspects in GSM. The maximum interference power that can be experienced by a victim GSM device for correct operation is given by:

(2) $$I_{\max}=P_{sens}+COR-9\;dB$$

Using the above formula one defines the minimum interfering signal attenuation *L_min_* required for interference-free operation (where ERP stands for the Equivalent Radiated Power and is the power measured on the antenna output. Here, it is the power of the 1st harmonic—the source of disturbances):

(3) $$L_{\min}=ERP-I_{\max}$$

Eventually, the formula for a safe distance between the GSM modem used in the sensor network and the victim terminal or BTS is defined by:

(4) $$d_{safe}[m]=10\exp\left(\frac{L_{\min}-2.15-20\log f_{MHz}}{20}\right)$$

In typical scenarios, *d_safe_* from terminals is on the order of ca. 30 m. As for the base stations, this distance varies with a BTS type between a few hundred meters for “normal BTS” down to a few tens of meters for “Pico BTS P1”.

### 3.3. Performance Issues—Facts and Myths

The wireless technologies presented in former sections are well fitted to sensor network applications, however they also possess weaknesses that will now be addressed. They can be considered as limiting factors and need to be considered by a sensor network designer in order not to overestimate the final sensor network performance.

As mentioned in Section 2.1.1, one of the attractive features of the ZigBee technology is its ability to self-organize and transmit sensor data by sending them through multiple intermediate ZigBee devices to a destination (e.g., a GSM modem). This advantage, however, comes at price of deteriorated transmission performance, which will be briefly discussed in this section. Two deciding factors for evaluating a communication system performance are the throughput (or data rate) and the packet delivery delay. While the former is rather obvious, the latter gains on importance in time-critical applications where the sensor data need to be effectively transferred within constrained time bounds.

**Figure 13:**
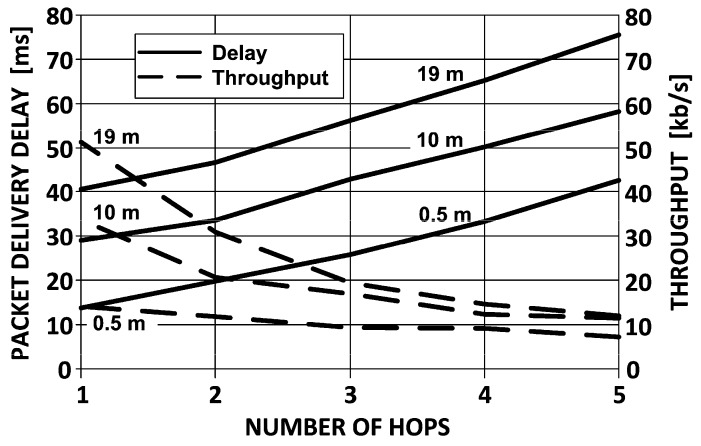
Measured ZigBee performance under multi-hop conditions: throughput and packet delivery delay.

Results of throughput measurements are shown in Figure 13 (dashed line) [23]. The most significant observation is the peak throughput obtained for the most closely separated devices (the upper curve corresponding to the distance of 0.5 m). In the best case, as it appears, the ZigBee devices offer the throughput of little above 50 kb/s which decreases exponentially down to merely 20% of the initial value, as the transmission hops five times. With devices more further apart (like 10 or 19 m), the throughput degradation is not that drastic and the final value after five hops is still a few kb/s, but the initial throughput in point-to-point connection (i.e., one hop) is limited to only several kb/s. It is by far much less than could be expected from IEEE 802.15.4 specification underlying ZigBee technology and which states that the data rate in 2.4 GHz ISM band equals 250 kb/s.

There are several reasons for this discrepancy, the most important of which are: first, the need to send, beside the measured data, also a constant volume of signaling information; and second, the need to compete for access to each of the intermediate nodes (in a multi-hop scenario as presented here) with other nodes attempting to transmit their sensor data these intermediate nodes as well.

As concerns the other performance factor—the packet delivery delay (as is shown in Figure 13) is linearly dependent on the distance between devices, approximately 15 ms every 10 meters of distance.

## 4. Power Supply in WSN

One has to remember that both sensors and detecting systems of chemical compounds and compositions require specified maintenance (like frequent exchange of filters, individual chemical analysis of samples in a lab, etc.). They usually consume a lot of electrical energy used for heating, detection and processing. A summary of the measured power consumption expected from different sensors has been presented in Table 3.

Considering autonomous supply for the monitoring system the photovoltaic battery set was found to be the most convenient. However, the size and efficiency of the solar panel must be carefully selected depending on:

- sensor types predicted for use (power consumption);
- frequency as well as the accepted measurement duration; and
- frequency of data transmission from the concentrator to the server.

Electrical power consumption of GSM/GPRS modem employed (Q2687H) is equal to 4.2 W.

**Figure 14:**
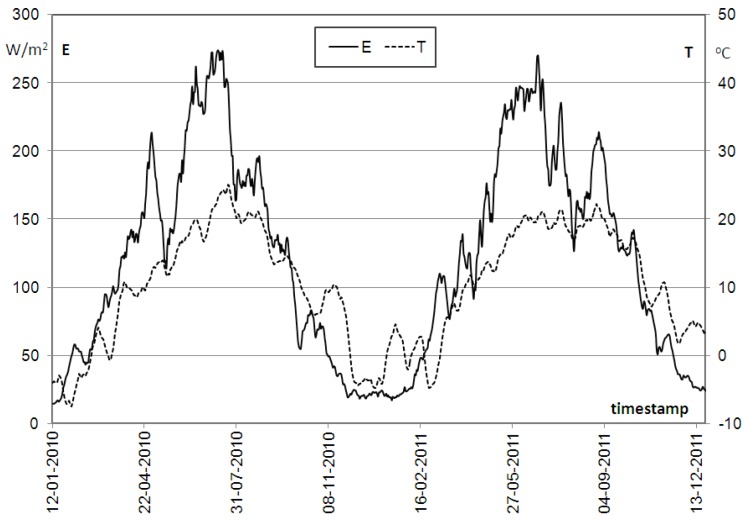
Mean value (for two weeks) of the solar irradiance E and temperature T versus time.

Before selection of the photovoltaic source, one has to recognize both temperature and irradiance (power incident on a surface) conditions in the area where the system is to be located. Therefore, respective measurements were performed for a period of two whole years, with particular attention to the most inconvenient conditions in the wintertime. Figure 14 presents the sun irradiance versus time over two years (2010/2011) of measurements as an example, for an area located at the campus of Wroclaw University of Technology.

The plots of *E(t)* and *T(t)* represent rolling means for 14 day-long periods. One can notice that the worst both irradiance and temperature conditions are usually observed in wintertime, which, in 2010/2011, was between mid-November and mid-February.

Therefore, in those months, the irradiance cannot provide enough energy to the system and to the battery used as a back-up source. Besides, the battery capacity was also decreased then. Concluding, the selection of both the solar panel size and the battery capacity has to be adapted to the worst environmental conditions. For the analyzed time (i.e., November–February), the rolling mean value of the ambient temperature was around −7 °C. Therefore, when using for example KYOCERA solar module, a decrease of efficiency by about 16% has be taken into account. In order to reduce unacceptably high levels of energy consumption, appropriate energy-saving modes in the system operation must be carefully considered. These modes should also affect sensors and the GSM/GPRS modem [24,25,26,27,28,29].

## 5. Conclusions

Multiple aspects discussed in this article can be summarized in the following few points:

- Wireless and wired sensor network have been recognized to play a key role in evaluating the holistic state of environment conditions as they allow measuring and transmitting various physical and chemical factors simultaneously for later combination, processing and forecasting. A general structure of a wide-area sensor network should include the local segment directly interfacing the sensor level, the core segment for conveying information over cellular backhaul and the segment for storing and processing the measurement data. A recommendable technique to use in the local segment is ZigBee (based on IEEE 802.15.4 specification), which offers self-organization properties and energy-saving features for low-data rate transmissions of sensor data. If available, cable (Ethernet) connections can also be used. In the core segment, one may consider cellular technologies for their wide-spread coverage and availability. These include the 2nd systems (such as GSM) as well as techniques cumulatively called “B3G” (Beyond 3rd Generation), including GPRS, EDGE, UMTS and LTE (Advanced) systems. All data measured and then transferred through the local and core segment require efficient storage mechanisms to be employed. A MySQL database cluster allowing reliable replication features is therefore necessary, such as one based on Galera solution where any action performed on data on one server is immediately replicated on all other backup servers so that a Single Point of Failure situation is minimized;
- Although radio techniques offer the greatest flexibility, scalability and ease of deployment, they are also prone to receive interference from other radiating devices on one hand and cause interference to other systems on the other hand. In the local segment, the greatest threat to ZigBee operation is posed by other short-range systems operating in 2.4 GHz ISM band. Fortunately, a few meter separation is usually enough to prevent any influence of these systems on ZigBee. In the core segment, the cellular system itself protects its devices from intra-system interference by implementing effective multiple-access schemes. As for other accidental devices that might cause disturbances, they are legally prohibited from running in licensed bands. Therefore, the EMC analysis should be focused on the potential impact that the concentrator (as a basic core segment component) may exert on other legacy GSM devices by unwanted radiated emissions (e.g., the 1st harmonic signals). These victim devices include base stations and other users’ terminals. Since their susceptibility to co-channel interference is well known, safe operating distances can be calculated between them and the concentrator for seamless operation;
- Besides EMC aspects, one should also consider technical limitations of the wireless techniques that are responsible for deteriorating their transmission properties. In ZigBee, the throughput and the packet delivery delay is mostly affected by the signaling overhead of transmission frames and the multi-hop operation. On the cellular system part, the major problem may arise from gaps in coverage. One should also consider appropriate system synchronization in real time for proper labeling of measured samples;
- The use of renewable energy sources in sensor systems is desirable and preferable at the moment. The application of an alternative electric energy supply requires power budget to be carried out with respect to both the sensor energy consumption and the power supply effectiveness under harsh environmental conditions

## Figures and Tables

**Table 1:** Channels definitions in ZigBee technology (IEEE 802.15.4).

Frequency Band (MHz)	Channels (*k*)	*k*-th Channel Center Frequency (MHz)
868.0–868.6	*k* = 1	*f_k_* = 868.3
902–928	*k* = 1,…, 10	*f_k_* = 906 + 2(*k* − 1)
2400–2483.5	*k* = 11,…, 26	*f_k_* = 2405 + 5·(*k* − 11)

**Table 2:** A simplified business model for the core segment technologies.

Item	Expected Maximum Price	Type
MySQL commercial license with database	*2000–10,000 USD*	Application. Annual subscription with full support
NGNIX server	*2000–4000 USD*	Application. Annual subscription with full support
Web servers	*2000–6000 USD*	Hardware multipurpose servers
SIM cards	*<10 USD*	Example price for: 1 GB/month/1 SIM card Bulk purchase based on a Service Layer Agreement (SLA) with a telecom operator

**Table 3:** Selected sensor parameters.

Measured Quantity	Type	Power Consumption (mW)
Temperature and humidity	SHT75	0.36
Carbon monoxide (CO)	TGS2442	46.8
Carbon dioxide (CO_2_)	TGS4161	600
Air contaminants (Ethanol, Toluene, Hydrogen)	TGS2602	720
Ammonia (NH_3_)	TGS2444	186
Diesel Engine Exhaust Gas (NO, NO_2_)	TGS2106	1080
Organic Solvent Vapors (Ethanol, Benzene, Methane)	TGS823	1680
Sulfur dioxide (SO_2_)	SO2-AE	3
Atmospheric pressure	MPX5100A	84
